# Nature-Based Therapy: Exploring the Processes That Foster Change

**DOI:** 10.3390/bs16040574

**Published:** 2026-04-11

**Authors:** Jessica D. Rhodes, Helen M. Boylan

**Affiliations:** 1 Department of Psychology & Neuroscience, Westminster College, New Wilmington, PA 16172, USA; 2 Harms Center for the Environment, Westminster College, New Wilmington, PA 16172, USA; boylanhm@westminster.edu

**Keywords:** nature therapy, mindfulness, cognition, mood, activity

## Abstract

A growing body of research has demonstrated the positive impact of nature-based interventions on cognition, affect, and overall well-being. However, important questions remain regarding the processes through which nature therapy exerts its effects. Participants (n = 188) were recruited and randomly assigned to one of four experimental conditions (nature therapy, nature only, mindfulness only, and control condition). While exposure to nature alone, mindfulness alone, and the combination of the two significantly decreased negative mood, only the combination of nature and mindfulness significantly improved positive mood. The present findings highlight the unique impact that nature-based interventions that also incorporate mindfulness techniques have on positive mood. These findings are consistent with research that demonstrates that positive mood enhancement is clinically meaningful.

## 1. Introduction

Over the past several decades, a growing body of research has highlighted the restorative influence of natural environments on human cognition, emotion, and overall psychological well-being (Bowler et al., 2010; Shuda et al., 2020). Often referred to as nature therapy, ecotherapy, or nature-based interventions, these practices broadly encompass structured and unstructured interactions with natural settings, including exposure to green spaces, guided outdoor activities, horticultural therapy, forest bathing, and wilderness programs. Although the specific modalities vary, they share the underlying assumption that humans possess an inherent affinity for the natural world and that reconnecting with these environments offers measurable cognitive and affective benefits (Bratman et al., 2015).

The theoretical foundations for nature therapy stem from two influential frameworks. Attention Restoration Theory (ART) proposes that natural landscapes attract attention in a restorative manner, allowing top-down cognitive processes the opportunity to recharge (Berman et al., 2008; Ohly et al., 2016). This is based on the premise that directed attention is limited and its usage can lead to “cognitive drain”. In other words, being immersed in nature can aid in restoring the “drain” that has occurred as a result of over-usage. The Stress Reduction Theory (SRT) developed by Ulrich et al. (1991) is an evolutionary perspective based on the idea that humans are bound to the natural world, finding the features of nature implicitly calming. SRT posits (Rowley et al., 2022) that stress is elevated among individuals who reside in urban surroundings because of their lack of connection with nature. When these individuals are reconnected with nature, it results in renewed positive emotions. Unlike ART, which emphasizes an active cognitive process, SRT posits that nature has a more passive effect on physical and psychological processes (Mason et al., 2022). Together, these perspectives suggest that nature therapy can produce both cognitive improvements, such as enhanced working memory, attentional capacity, and cognitive flexibility, and affective enhancements, including reduced stress, improved mood, and greater emotional regulation.

Empirical findings and broad reviews conclude that exposure to natural environments and structured nature-based interventions produce benefits for cognition and mental health, supporting the claims of ART and SRT theoretical models (Bowler et al., 2010; Djernis et al., 2019; Siah et al., 2023). Studies consistently show that even brief contact with natural environments can improve performance on tasks that require attention and working memory. For example, experimental lab and field studies report that even a brief nature exposure (e.g., walks or viewing natural scenes) results in improved attention and working memory in subjects (Berman et al., 2008). Nature-based interventions have also been associated with reductions in symptoms of anxiety and depression, as well as decreased rumination and lower perceived stress (Bratman et al., 2015; Shanahan et al., 2016; Twohig-Bennett & Jones, 2018). Importantly, these benefits appear across developmental groups and clinical populations, including individuals with mood disorders, attentional difficulties, and trauma histories (Coventry et al., 2021; Hyvönen et al., 2023; Yang et al., 2023). Emerging research also highlights the potential for nature therapy to strengthen resilience, promote mindfulness, and enhance overall psychological flourishing (White et al., 2023).

Despite these promising findings, important questions remain regarding the processes through which nature therapy exerts its effects and the conditions under which therapeutic benefits are maximized. Mindfulness, a stress-reduction and mood-enhancing strategy, is often included in nature-based interventions and may play an important role in its observed benefits. Mindfulness practice has been associated with improvements in interpersonal and intrapersonal abilities, as well as emotion regulation (Davis & Hayes, 2011). Mindfulness practice has also been demonstrated to produce positive results in both clinical (Keng et al., 2011) and non-clinical (Chiesa & Serretti, 2009; Dane & Brummel, 2014) populations. For example, mindfulness-based approaches have been used to treat anxiety and depression (Hofmann et al., 2010; Kuyken et al., 2016).

The present study seeks to clarify the important processes underlying nature-based therapeutic effects. Specifically, it evaluates the independent and combined contributions of nature exposure and mindfulness practice to cognitive functioning and affective well-being. By isolating these components, this work aims to advance a more nuanced understanding of the processes through which nature therapy operates and to inform the development of more targeted, evidence-based intervention strategies.

## 2. Materials and Methods

All methods and procedures were approved by the Westminster College Institutional Review Board. Participants (n = 188) were recruited through Westminster College Introduction to Psychology courses, Introduction to Liberal Arts Education courses, and convenience sampling. Informed consent was provided, and all participants were required to be eighteen or older to participate in the study. Following informed consent, participants completed the study procedures (details provided below). Participants received remuneration in the form of a ten-dollar gift card to a local establishment or research participation credit for Introduction to Psychology at the discretion of their instructor.

Backward Digit Span. Cognition (specifically, attention and working memory) was measured utilizing a backward digit span. A digit span task is a measure of verbal short-term working memory. Strings of numerical digits, three to nine digits in length, dictated at a rate of one digit/second were pre-recorded and presented to participants for testing. Following each string, participants were asked to record the digits within the string in backward order. For example, if “2 7 5” was presented, participants would record “5 7 2” on their participant record form. Following instructions and an opportunity to ask questions, participants were presented with a brief practice round, then two administrations of each span beginning with a span of three digits and increasing sequentially up to nine digits. The primary dependent variable utilized for analysis was number of correct trials. The backward digit span task is an appropriate and well-established measure of working memory capacity, particularly indexing the manipulation component of executive functioning (Hilbert et al., 2015).

Brief Mood Introspection Scale (BMIS). Mood was measured using the Brief Mood Introspection Scale (Mayer & Gaschke, 1988). Participants answer the extent to which 17 adjectives or phrases describe their present mood using a four-point Likert Scale (1 = definitely do not feel, 2 = do not feel, 3 = slightly feel, 4 = definitely feel). For example, participants must indicate how much they feel “lively” at the present time. From this scale, negative mood and positive mood scores were calculated. In the present sample, both the pre-and post-manipulation assessment of the BMIS demonstrated adequate reliability (Spearman–Brown coefficients = 0.72, 0.71, respectively).

Participants were randomly assigned to one of the four conditions detailed in Table 1: nature therapy, mindfulness only, nature only, and a control condition. Participants were recruited from a single pool of undergraduate students throughout the study period using consistent recruitment and eligibility procedures. While the control condition was introduced later in the recruitment period, enrollment into the active arms was still ongoing at the time. Therefore, once the control condition was added, participants were randomized with equal likelihood across all four study conditions. This timing difference is acknowledged as a potential limitation with respect to internal validity and possible cohort effects. After finishing the pre-manipulation assessments, participants completed a 20-min gentle walk in their assigned condition (with the exception of the control condition), which was guided by a research assistant. Upon completion of the manipulation (i.e., walk or seated educational session), participants completed the post-manipulation assessments and were debriefed and remunerated.

The walking route and stopping points were identical across the two outdoor conditions (nature therapy and nature only) and took place on a paved walking trail on campus in the presence of a moderate level of nature that included trees, a stream, and green space. The mindfulness indoors condition took place inside the college library and was timed to match the duration and stops of the outdoor conditions. Scripts and mindfulness procedures were standardized to ensure comparable timing across all conditions and excerpts of each script are provided in Table 2. As such, the mindfulness intervention is considered a guided concentration exercise or formal practice (Crane et al., 2017; Djernis et al., 2019). In the control condition, which took place in a classroom without windows, the same educational script used in the nature only condition was presented with a slideshow highlighting the various environmental topics. To maintain participants’ directed attention and to mimic the structure of the walking conditions, multiple-choice quiz questions were embedded in the slideshow at time points corresponding to the walking between stops in the other conditions.

## 3. Results

Table 3 provides basic demographic information across the four groups; there were no significant group differences. Repeated measures ANOVAs exploring changes in the primary dependent variables (cognition, mood) over time (pre-test, post-test) were performed to evaluate the effect of group (experimental manipulation). The means and standard deviations for the dependent variables are presented in Table 3. Table 4 provides pre- and post-test means and standard deviations across groups.

### 3.1. Cognition

While there was a main effect of time on cognition, F (1, 181) = 34.15, *p* < 0.001, the Time × Group interaction was not significant, F (3, 181) = 1.01, *p* = 0.39, suggesting that across all groups, participants’ digit span scores improved from pre- to post-test (*p*’s < 0.03, *η*^2^*_p_*’s < 0.12).

### 3.2. Negative Mood

There was a significant main effect of time on negative mood, F (1, 184) = 37.43, *p* < 0.001, as well as a significant Time × Group interaction, F (3, 184) = 8.59, *p* < 0.001. Follow-up analyses demonstrated that there was a significant decrease in negative mood across pre- and post-test assessment for all groups (*p*’s < 0.003, nature only *η*^2^*_p_* = 0.26, nature therapy *η*^2^*_p_* = 0.26, mindfulness *η*^2^*_p_* = 0.05), except the control condition (*p* = 0.61, *η*^2^*_p_* = 0.03). There were no significant group differences in pre-test scores of negative mood (*p*’s > 0.26).

### 3.3. Positive Mood

Although there was not a significant main effect of time on positive mood, F(1, 184) = 1.66, *p* = 0.20, there was a significant Time × Group interaction, F(3, 184) = 3.54, *p* = 0.02, *η*^2^*_p_* = 0.06. Follow-up analyses demonstrated that while there was no significant difference from pre-test to post-test measurements in the nature only (*p* = 0.81) and mindfulness only (*p* = 0.11) groups, the control condition demonstrated a marginal decrease in positive mood across assessments (*p* = 0.06, *η*^2^*_p_* = 0.02) and the nature therapy group demonstrated a significant increase in positive mood across the manipulation (*p* = 0.04, *η*^2^*_p_* = 0.02). With the exception of a significant pre-test difference between the nature therapy group and the mindfulness only group (*p* = 0.04), pre-test levels of positive mood did not significantly differ across groups (*p*’s > 0.12). While the omnibus analysis indicated significant between-group differences in positive mood change, F(3, 184) = 3.38, *p* = 0.02, *η*^2^ = 0.05, post hoc comparisons suggested that the combined condition demonstrated a marginally greater increase relative to the mindfulness and control groups (*p*’s = 0.07) and did not significantly differ from the nature condition.

## 4. Discussion

The present study is the first to our knowledge to directly and systematically evaluate the processes of nature therapy that are related to change in emotion and cognition. Results indicated that while exposure to natural settings alone, mindfulness alone, and the combination of the two significantly decreased negative mood, the combined nature therapy condition showed a trend toward greater improvement in positive mood relative to mindfulness and control conditions, although it did not significantly differ from the nature-only condition. These findings have important therapeutic implications.

Enhancing positive mood has increasingly been recognized within clinical psychology as an important treatment target in its own right, rather than merely the opposite of reducing negative affect. A substantial body of research demonstrates that positive affect plays a protective role in mental health by enhancing cognition, supporting adaptive coping, and facilitating engagement in rewarding activities (Fredrickson, 2001; Garland et al., 2010). Low positive affect, by contrast, is a core feature of depression and other internalizing disorders and predicts poorer treatment response (Dunn, 2012). Interventions that successfully increase positive emotions, such as behavioral activation, positive psychology exercises, and mindfulness-based treatments, have been shown to improve psychological functioning, enhance resilience, and promote and maintain recovery (Craske et al., 2016; Sin & Lyubomirsky, 2009). Thus, positive mood enhancement is regarded as a clinically meaningful mechanism of change, highlighting the relevance of the present findings that nature-based interventions combined with mindfulness increase positive affect.

The absence of significant within-group differences in positive mood in the nature-only condition was somewhat surprising and not consistent with a large body of prior literature demonstrating that exposure to natural environments is associated with improvements in mood and emotional well-being (Bratman et al., 2012; Capaldi et al., 2014; Liu et al., 2024) and thus warrants further consideration. The intensity of nature exposure in the present study was relatively limited (i.e., natural context on a college campus) and is consistent with a semi-natural environment (Mausner, 1996). The prior literature demonstrating robust improvements in positive mood typically involves more immersive or prolonged exposure to natural environments, with evidence suggesting that extended or more intensive nature experiences yield stronger restorative benefits than brief or low-dose exposures (Pham & Sanocki, 2024; White et al., 2019). As such, the present findings may represent a conservative estimate of the potential impact of nature exposure.

It is also important to consider the role of attentional engagement. The guided mindfulness strategy may have functioned as an attentional scaffold, helping participants engage with the environment (Kabat-Zinn, 2003). This may be particularly important in lower-dose (such as in the present study) or urban settings, where natural environments provide fewer intrinsically restorative cues/stimuli and may not automatically elicit the effortless attention (“soft fascination”) described in Attention Restoration Theory (Djernis et al., 2019; Ohly et al., 2016). In contrast, in more immersive natural contexts, less formal/guided mindfulness strategies, or passive exposure, may be sufficient to elicit significant changes in positive mood (Kaplan, 2001). Future research ought to consider varying both the intensity of nature exposure and the degree of mindfulness formality to clarify these relationships.

Although novel, this study is not without limitations. First, the relatively small sample size, particularly in the control condition, and lack of diversity may have limited statistical power to detect more subtle effects, increased susceptibility to sampling variability and outliers, and constrained the generalizability of the findings. Replication with larger and more balanced groups and diverse samples is needed to strengthen confidence in the observed patterns. Second, although the control condition was added later in the recruitment process, all participants were recruited from the same source population of undergraduate students using identical eligibility criteria and recruitment procedures. Importantly, recruitment for the active intervention conditions remained ongoing at the time the control arm was introduced, such that all subsequently enrolled participants had an equal probability of assignment to any of the four study conditions. Nevertheless, the later addition of the control group represents a potential limitation, as differences related to timing of enrollment or unmeasured cohort effects may pose a threat to group comparability and internal validity. Accordingly, findings involving the control condition should be interpreted with appropriate caution. Third, the study relied on the backward digit span task as the primary measure of attention/cognition. Repeated exposure to digit span tasks has been demonstrated to yield performance improvements (Holm et al., 2022) due to increased familiarity with task demands, strategy, and reduced cognitive load over time (Conway et al., 2005; Waters & Caplan, 2003). As such, the uniform improvement observed across conditions in the present study may reflect general testing effects, which may obscure between-group differences. Although this task is widely used and assesses aspects of working memory and attention, it may not be sufficiently sensitive (Arnell et al., 2010) to the specific processes theorized to be influenced by nature exposure, such as involuntary attention capture or directed-attention restoration (Ohly et al., 2016; Stevenson et al., 2018). As a result, the present findings may reflect task limitations rather than the across-group cognitive change. Future research would benefit from incorporating a broader battery of measures that isolate sustained, selective, and executive components of attention to more precisely evaluate the cognitive mechanisms through which nature-based interventions exert their effects. It is also important to note that a substantial body of research demonstrates that physical activity is independently associated with positive improvements in both mood and cognition (e.g., Chang et al., 2012; Josefsson et al., 2014; Reed & Buck, 2009). As such, it is possible that the presently observed mood benefits, both positive and negative, may be attributable, at least in part, to the effects of physical activity rather than to the experimental manipulation specifically. Because physical exertion was not directly measured or controlled, the contribution of activity represents a potential confounding variable and should be more rigorously accounted for in future research.

It is also important to note that the mindfulness practice implemented in the present study would be considered guided and more formal in nature (Crane et al., 2017; Djernis et al., 2019). It is possible that informal or more naturalistic mindfulness practice, such as those that are integrated into an individual’s daily routine, may produce different outcomes (e.g., Petrovic et al., 2025) than those observed in the present study, which represents an important future direction. However, the structured nature of the present protocol may foster replication and feasibility within formal clinical settings. As such, the present findings may be especially relevant for treatment contexts, while future research ought to explore how varying levels of formality impact both effectiveness and applicability. Similarly, given findings that novice practitioners of mediation/mindfulness may experience greater attentional effort/demand during initial training and that rich natural environments may offset those demands (Lymeus et al., 2017, 2018), it is important for future investigations to explore whether baseline mindfulness/meditation experience moderates responses across conditions.

In sum, the present study provides novel insight into and advances understanding of the change-related processes of nature-based interventions by directly comparing the cognitive and affective effects of mindfulness practice, nature exposure, and their combination. Although participants in all three active conditions demonstrated reductions in negative affect over time, only the nature therapy condition demonstrated a statistically significant within-group increase in positive mood from pre- to post-test, suggesting that the combination of guided mindfulness practice and exposure to natural environments may confer additional benefits for positive emotional functioning beyond reductions in distress. The results of the present study also suggest that the combination of mindfulness-based practice in natural environments may offer affective benefits beyond those achieved through mindfulness alone and highlight the importance of incorporating natural settings into therapeutic practices aimed at promoting positive emotional changes. By identifying the specific components of nature therapy that drive improvements in emotional functioning, this study offers a clearer foundation for refining intervention strategies and advancing theory on how natural environments support psychological health. Future research utilizing larger samples, multimethod assessments, and diverse populations will be essential for further clarifying the pathways through which nature-based practices promote both relief from distress and the cultivation of positive emotional experiences.

## Figures and Tables

**Table 1:** Experimental Conditions.

	Group 1 Nature Therapy	Group 2 Mindfulness Only	Group 3 Nature Only	Group 4 Control Condition
Description	Mindfulness in nature	Mindfulness indoors	Educational hike in nature	Educational setting indoors
Location	Walking trail	Library	Walking trail	Indoor classroom
Manipulation	Four outdoor “stops” with mindfulness focusing on a different sense at each stop; gentle walking between each stop	Four indoor (no exposure to windows) “stops” with mindfulness focusing on different senses at each stop; gentle walking between each stop	Four outdoor “stops” with information about environmental science being read at each stop; gentle walking between each stop	While seated in a windowless classroom, participants received information about environmental science

**Table 2:** Script Excerpts.

Condition	Script Excerpts
**Nature Therapy**	Here is our second stop. Get into a comfortable standing position. In this area, we are going to focus on the sounds of the stream. (Pause) Listen very carefully to the sounds of nature. (Pause) Maybe you can hear gurgling of the water. (Pause) Maybe you can hear the rushing sound as the water moves along the rocks. (Pause) Focus carefully on the sounds. (Pause) As you are trying to focus, your mind may wander. That is ok. Bring your mind back to focusing on the sounds. We are now going to spend one quiet minute focusing on the sounds of this area. (Pause) Now that our minute is complete, bring your awareness back to all of your senses. Take a few deep breaths. (Pause)
**Mindfulness Only**	Here is our second stop. Get into a comfortable standing position. In this area, we are going to focus on the sounds of the area. (Pause) Listen very carefully to the sounds of the indoors. (Pause) Maybe you can hear the heating and cooling system. (Pause) Maybe you can hear the creaks and movement of the building. (Pause) Focus carefully on the sounds. (Pause) As you are trying to focus, your mind may wander. That is ok. Bring your mind back to focusing on the sounds. We are now going to spend one quiet minute focusing on the sounds of this area. (Pause) Now that our minute is complete, bring your awareness back to all of your senses. Take a few deep breaths. (Pause)
**Nature Only & Control Condition**	[Here at this stop (omitted in the Control Condition script)], I will talk about the science behind streams. A stream is a continuous body of surface water flowing within the bed and banks of a channel. Depending on its location or certain characteristics, a stream may be referred to by a variety of local or regional names. Long large streams are usually called rivers, while smaller, less voluminous and more intermittent streams are known as streamlets, brooks or creeks, etc. The flow of a stream is controlled by three inputs—surface runoffs (from precipitation or meltwater), daylighted subterranean water, and surfaced groundwater (spring water). The surface and subterranean water are highly variable between periods of rainfall. Groundwater, on the other hand, has a relatively constant input and is controlled more by long-term patterns of precipitation. The stream encompasses surface, subsurface and groundwater fluxes that respond to geological, geomorphological, hydrological and biotic controls. Streams are important as conduits in the water cycle, instruments in groundwater recharge, and corridors for fish and wildlife migration. The biological habitat in the immediate vicinity of a stream is called a riparian zone. Given the status of the ongoing Holocene extinction, streams play an important corridor role in connecting fragmented habitats and thus in conserving biodiversity. The study of streams and waterways in general is known as surface hydrology and is a core element of environmental geography.

**Table 3:** Descriptive Statistics for Demographic and Primary Variables.

	Nature Therapy (n = 60)	Mindfulness (n = 57)	Nature (n = 60)	Control (n = 11)	*p*-Value
Age (Mean, SD)	19.48 (1.14)	19.09 (1.14)	19.13 (1.11)	19.45 (0.93)	0.19
Gender (% Female)	62%	58%	48%	55%	0.24
Race (% White)	93%	90%	93%	100%	0.63
Digit Span Change (Mean, SD)	22.77 (36.55)	16.66 (36.64)	43.22 (89.64)	26.35 (35.30)	0.10
Positive Mood Change (Mean, SD)	4.31 (13.85)	−1.59 (12.88)	1.10 (11.48)	−6.14 (8.21)	0.02
Negative Mood Change (Mean, SD)	−15.90 (16.62)	−5.99 (17.84)	−16.73 (16.97)	4.34 (12.94)	<0.001

**Table 4:** Group Differences for Cognition, Negative Mood, and Positive Mood.

	Cognition			Negative Mood			Positive Mood		
	Test	Mean (SD)	*p* Value	Test	Mean (SD)	*p* Value	Test	Mean (SD)	*p* Value
Nature Therapy	Pre	5.23 (0.26)	***	Pre	18.73 (0.55)	***	Pre	23.65 (0.51)	**
	Post	6.26 (0.27)		Post	15.68 (0.59)		Post	25.14 (0.52)	
Mindfulness	Pre	5.05 (0.26)	**	Pre	17.84 (0.56)	**	Pre	25.14 (0.52)	ns
	Post	5.67 (0.27)		Post	16.68 (0.61)		Post	25.54 (0.51)	
Nature	Pre	4.55 (0.26)	***	Pre	18.17 (0.55)	***	Pre	23.98 (0.51)	ns
	Post	5.70 (0.26)		Post	15.13 (0.59)		Post	24.07 (0.50)	
Control	Pre	5.18 (0.60)	**	Pre	17.55 (1.28)	ns	Pre	24.27 (1.19)	*
	Post	6.36 (0.61)		Post	18.00 (1.38)		Post	22.73 (1.17)	

*p* < 0.001 ***, *p* < 0.05 **, *p* < 0.10 *, ns = not significant.

## Data Availability

Data for this study are available upon request (please contact the authors).
